# Development and Validation of a Prognostic Nomogram for Hypopharyngeal Carcinoma

**DOI:** 10.3389/fonc.2021.696952

**Published:** 2021-06-21

**Authors:** Shu Tian, Qin Li, Ruichen Li, Xinyu Chen, Zhonghua Tao, Hongli Gong, Xiaoshen Wang, Xichun Hu

**Affiliations:** ^1^ Department of Medical Oncology, Fudan University Shanghai Cancer Center, Shanghai, China; ^2^ Department of Oncology, Shanghai Medical College, Fudan University, Shanghai, China; ^3^ Department of Radiation Oncology, Eye Ear Nose and Throat Hospital, Fudan University, Shanghai, China; ^4^ Fudan University Shanghai Cancer Center, Key Laboratory of Medical Epigenetics and Metabolism, Institutes of Biomedical Sciences, Fudan University, Shanghai, China; ^5^ Shanghai Key Clinical Disciplines of Otorhinolaryngology, Department of Otolaryngology, Eye Ear Nose and Throat Hospital, Fudan University, Shanghai, China

**Keywords:** hypopharyngeal carcinoma, nomogram, radiotherapy, surgery, prognosis, survival analysis

## Abstract

Hypopharyngeal squamous-cell carcinoma (HSCC) is a relatively rare head and neck cancer, with great variation in patient outcomes. This study aimed to develop a prognostic nomogram for patients with HSCC. From the Surveillance, Epidemiology, and End Results (SEER) database, we retrieved the clinical data of 2198 patients diagnosed with HSCC between 2010 and 2016. The patients were randomly assigned at a 4:1 ratio to the training set or the validation set. An external validation was performed by a set of 233 patients with locally advanced HSCC treated at our center. A Cox proportional hazards regression model was used to assess the relationship between each variable and overall survival (OS). Cox multivariate regression analysis was performed, and the results were used to develop a prognostic nomogram. The calibration curve and concordance index (C-index) were used to evaluate the accuracy of the prognostic nomogram. With a median overall follow-up time of 41 months (interquartile range: 20 to 61), the median OS for the entire cohort of SEER database was 24 months. The 3-year and 5-year OS rates were 41.3% and 32.5%, respectively. The Cox multivariate regression analysis of the training set showed that age, marital status, race, T stage, N stage, M stage, TNM stage, local treatment, and chemotherapy were correlated with OS. The nomogram showed a superior C-index over TNM stage (training set: 0.718 *vs* 0.627; validation set: 0.708 *vs* 0.598; external validation set: 0.709 *vs* 0.597), and the calibration curve showed a high level of concordance between the predicted OS and the actual OS. The nomogram provides a relatively accurate and applicable prediction of the survival outcome of patients with HSCC.

## Introduction

Hypopharyngeal carcinoma is relatively rare and accounts for only approximately 3% of all head and neck tumors (1, 2). Approximately 95% of hypopharyngeal tumors are squamous-cell carcinoma (2). Hypopharyngeal squamous-cell carcinoma (HSCC) is often occult with atypical early symptoms due to its anatomical features, and approximately 80% of patients are already in stage III-IV at diagnosis (2, 3). A population cohort study of 2939 patients with hypopharyngeal carcinoma showed that 10.5% of patients were in tumor-node-metastasis (TNM) stage I, 12.1% were in stage II, 23.0% were in stage III, and 52.6% were in stage IV at diagnosis, with great variation in patient outcomes (3). The 5-year overall cancer-specific survival (CSS) rate is 33.4%, while the rate is 63.1% for stage I, 57.5% for stage II, 41.8% for stage III, and 22% for stage IV (3). However, the widely used American Joint Committee on Cancer (AJCC) TNM staging system remains has some limitations to assess prognosis in clinical practice. The outcomes of HSCC are also related to many clinical parameters, such as age (> 70 is an adverse prognostic factor) and primary site (piriform sinus tumor is associated with more favorable outcomes, followed by postcricoid region and then posterior pharyngeal wall) (4, 5). Treatment modalities probably affect patient survival, but the conclusions differ across studies (6–8). However, there is a lack of prognostic scoring systems that take those above clinical factors into account.

Nomogram is a visual statistical tool and can improve predictive accuracy for survival outcomes of tumor patients in clinical practice (9, 10). Several studies have shown that nomograms are superior to the TNM staging system in predicting prognoses (11, 12). By combining multiple clinical and pathological factors, nomograms can be used to assess the survival outcome of individual patient. However, few studies have yet been developed a prognostic nomogram for HSCC. The Surveillance, Epidemiology, and End Results (SEER) database is an authoritative source of cancer prevalence and survival in the United States, as it covers approximately 28% of the US population (6). Therefore, the SEER database can provide many cases for the development of predictive models for tumors, especially rare tumors. In this study, we retrieved the clinical data *via* the updated SEER database, including demographics, clinicopathological parameters, and treatment modalities, and established a nomogram to predict prognostic outcomes of patients with HSCC. We also performed internal and external validation.

## Patients and Methods

### Patient Selection

We retrieved patient data from the updated SEER database (https://seer.cancer.gov), which included information on radiotherapy and chemotherapy (Incidence -SEER 18 Regs Custom Data with additional treatment fields, Nov 2018 Sub, 1975-2016 varying). We used SEER* Stat software (released: August 08, 2019, version 8.3.6; http://seer.cancer.gov/seerstat) to download the data. The screening criteria were as follows: 1) primary site: hypopharynx, which was coded as C12.9, C13.0, C13.1, C13.2, C13.8, or C13.9 according to the International Classification of Diseases for Oncology, Third Edition (ICD-O-3); 2) pathologically confirmed squamous-cell carcinoma, coded as 8050-8089 according to ICD-O-3; 3) complete follow-up data, including survival and cause of death; 4) a first primary tumor, confirmed in 2010 or later; and 5) detailed information on variables, including age, sex, marital status, race, insurance, and TNM stage at diagnosis, as well as treatment mode of the primary tumor, such as surgery, radiotherapy, and chemotherapy. In addition, for external validation, we selected patients with locally advanced HSCC who were treated in the Department of Radiation Oncology, Eye and ENT Hospital, Fudan University, between April 2014 and December 2017. In this study, HSCC patients from the SEER database and treated at our center were both staged according to the seventh edition of the AJCC TNM Cancer Staging Manual.

The HSCC cancer-specific survival and noncancer-specific survival were extracted from the SEER variables of cause-specific death classification and other cause-of-death classification. Information on surgery and radiotherapy was extracted from the following fields: radiation sequence with surgery, reason for no cancer-directed surgery, and radiation recode. Information on primary-site surgery was extracted from the field “RX Summ-Surg Prim Site”. Primary-site surgery was coded as 20-52 according to the 2018 SEER program coding and staging manual.

### Statistical Analysis

SPSS v22.0 (IBM, Armonk, NY, USA) and R for Windows v3.5.1 (https://www.r-project.org) were used for the statistical analysis. Categorical variables were analyzed using a chi-squared test. The Kaplan-Meier method and log-rank test were used for the survival analysis. A Cox proportional hazards regression model was used for the univariate and multivariate analyses to identify prognostic factors, and independent prognostic factors identified by the Cox multivariate analysis were used to develop the prognostic nomogram. The concordance index (C-index) and the Brier score were used to evaluate the performance of the prognostic nomogram, while the calibration curve was used for internal validation of the nomogram. We compared the predictive performance of the prognostic nomogram with that of TNM staging. We also performed a competing risk analysis because noncancer-specific death competed with cancer-specific death. All tests were two-sided, and P < 0.05 was considered statistically significant.

## Results

### Patient Characteristics

**Figure 1:**
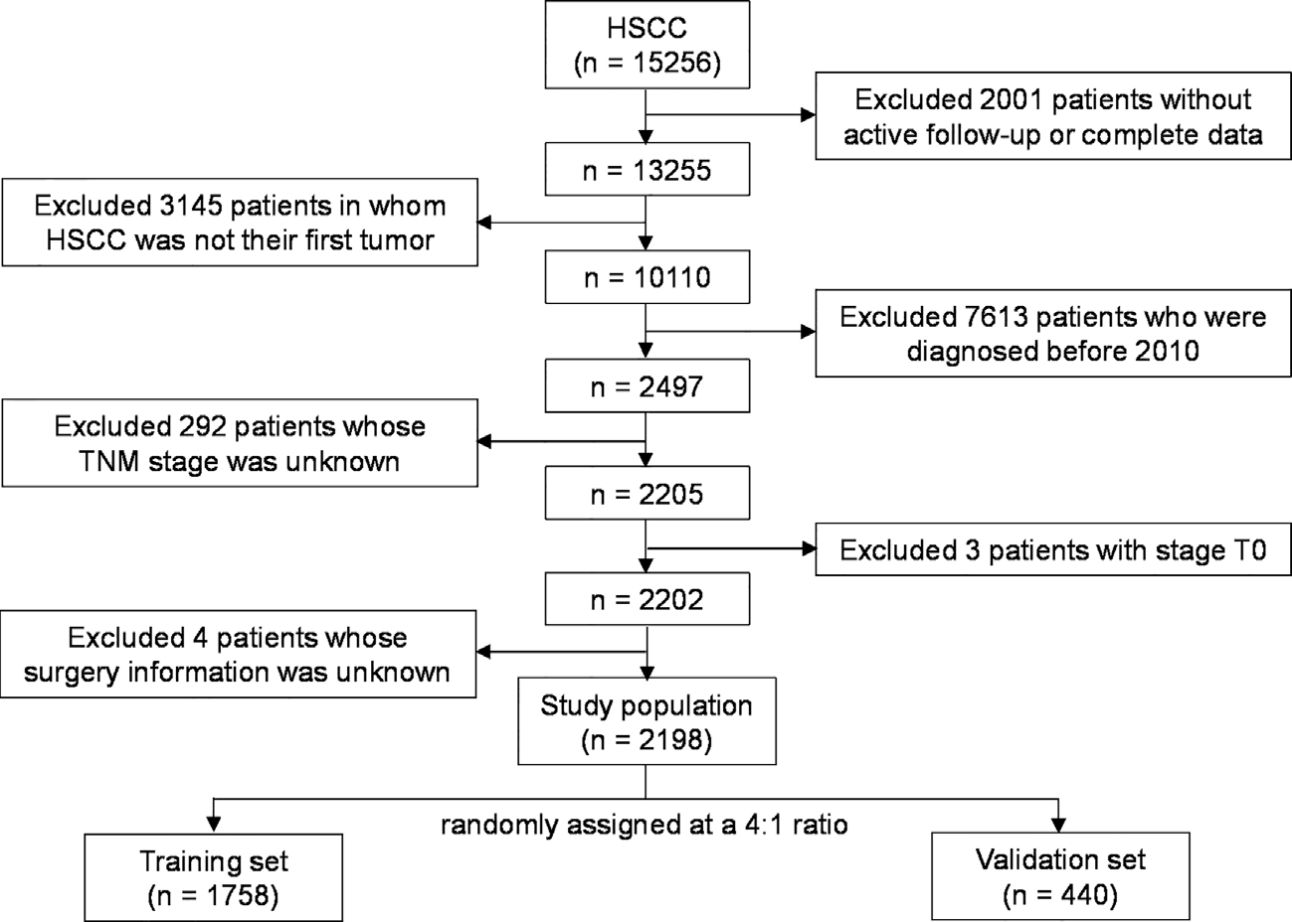
Screening process.

We identified a total of 15,256 patients who were pathologically confirmed to have HSCC between 1975 and 2016 in the SEER database. Of these patients, 2001 patients were excluded due to a lack of complete follow-up data, 3145 patients were excluded because HSCC was not their only tumor, 7613 patients were excluded because they were diagnosed before 2010 (no TNM staging information per the seventh edition of the AJCC Cancer Staging Manual), 292 patients were excluded due to unknown TNM stage, three patients were excluded because they were stage T0, and four patients were excluded due to unknown surgical details. Finally, 2198 patients were included in this study and were randomly assigned at a 4:1 ratio to the training set (n = 1758) or the validation set (n = 440). **Figure 1** illustrates the screening process. **Table 1** shows the demographics and clinical characteristics of the patients, 78.7% of whom were diagnosed with locally advanced HSCC (stages III-IVB). Moreover, 4.1% of the patients received surgery alone, 80.7% received radiotherapy alone, and 14.1% received both surgery and radiotherapy. The external validation set included 233 patients with locally advanced HSCC who were treated at our center, and **Table S1** shows their demographics and clinical characteristics.

**Table 1:** Demographics and clinical characteristics of the HSCC training and validation sets from the SEER database.

Characteristic	All patients	Training set	Validation set
	(n = 2198) No. (%)	(n = 1758) No. (%)	(n = 440) No. (%)
Age			
≤50 years	206 (9.4)	171 (9.7)	35 (8)
51-60 years	719 (32.7)	562 (32)	157 (35.7)
61-70 years	749 (34.1)	607 (34.5)	142 (32.3)
>70 years	524 (23.8)	418 (23.8)	106 (24.1)
Sex			
Male	1825 (83)	1466 (83.4)	359 (81.6)
Female	373 (17)	292 (16.6)	81 (18.4)
Marital status			
Married	942 (42.9)	749 (42.6)	193 (43.9)
Others	1256 (57.1)	1009 (57.4)	247 (56.1)
Race			
White	1640 (74.6)	1325 (75.4)	315 (71.6)
Black	391 (17.8)	302 (17.2)	89 (20.2)
Others	167 (7.6)	131 (7.5)	36 (8.2)
Insurance			
No/unknown	150 (6.8)	128 (7.3)	22 (5)
Yes	2048 (93.2)	1630 (92.7)	418 (95)
Primary site			
Pyriform sinus	1272 (57.9)	1002 (57)	270 (61.4)
Postcricoid region	58 (2.6)	40 (2.3)	18 (4.1)
Posterior pharyngeal wall	151 (6.9)	127 (7.2)	24 (5.5)
NOS	717 (32.6)	589 (33.5)	128 (29.1)
Grade			
Well differentiated	82 (3.7)	61 (3.5)	21 (4.8)
Moderately differentiated	906 (41.2)	722 (41.1)	184 (41.8)
Poorly differentiated	728 (33.1)	587 (33.4)	141 (32)
Undifferentiated	19 (0.9)	10 (0.6)	9 (2)
Unknown	463 (21.1)	378 (21.5)	85 (19.3)
T stage			
T1	217 (9.9)	173 (9.8)	44 (10)
T2	778 (35.4)	626 (35.6)	152 (34.5)
T3	552 (25.1)	438 (24.9)	114 (25.9)
T4a	443 (20.2)	350 (19.9)	93 (21.1)
T4b	208 (9.5)	171 (9.7)	37 (8.4)
N stage			
N0	546 (24.8)	428 (24.3)	118 (26.8)
N1	401 (18.2)	333 (18.9)	68 (15.5)
N2	1124 (51.1)	894 (50.9)	230 (52.3)
N3	127 (5.8)	103 (5.9)	24 (5.5)
M stage			
M0	2016 (91.7)	1615 (91.9)	401 (91.1)
M1	182 (8.3)	143 (8.1)	39 (8.9)
TNM stage			
I	81 (3.7)	56 (3.2)	25 (5.7)
II	204 (9.3)	160 (9.1)	44 (10)
III	396 (18)	321 (18.3)	75 (17)
IVA	1075 (48.9)	865 (49.2)	210 (47.7)
IVB	260 (11.8)	213 (12.1)	47 (10.7)
IVC	182 (8.3)	143 (8.1)	39 (8.9)
Surgery and radiotherapy			
Surgery	91 (4.1)	64 (3.6)	27 (6.1)
Radiotherapy	1777 (80.8)	1440 (81.9)	337 (76.6)
Surgery +radiotherapy	309 (14.1)	237 (13.5)	72 (16.4)
Both not given	21 (1)	17 (1)	4 (0.9)
Chemotherapy			
No/unknown	654 (29.8)	514 (29.2)	140 (31.8)
Yes	1544 (70.2)	1244 (70.8)	300 (68.2)

HSCC, hypopharyngeal squamous cell carcinoma; NOS, not otherwise specified; TNM, tumor-node-metastasis.

For the 2198 patients, the median follow-up time was 41 months (interquartile range: 20 to 61). The 3-year and 5-year OS rates were 41.3% (95% CI, 38.9% to 43.7%) and 32.5% (95% CI, 30.0% to 35.0%), respectively; the 3-year and 5-year CSS rates were 50.2% (95% CI, 47.7% to 52.7%) and 44.0% (95% CI, 41.3% to 46.7%), respectively. The median OS of all patients was 24 months, and the median survival was 24 months in the training set and 29 months in the validation set. For the external validation set, the median follow-up time was 27.9 months (interquartile range: 19.3 to 38.3), and the 3-year OS rate was 64.6% (95% CI, 56.6% to 72.6%).

### Construction of the Nomogram

**Figure 2:**
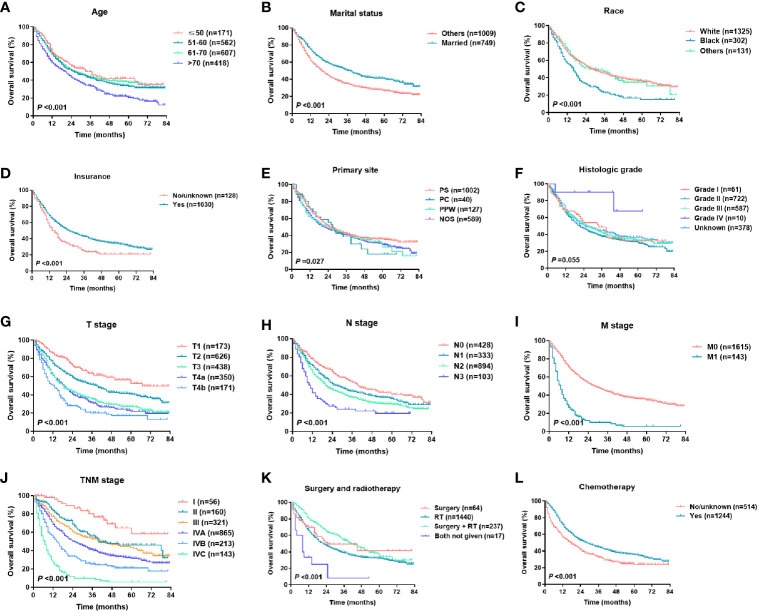
Kaplan-Meier survival curves of HSCC patients in different subgroups: **(A)** age, **(B)** marital status, **(C)** race, **(D)** insurance status, **(E)** primary site, **(F)** histological grade, **(G)** T stage, **(H)** N stage, **(I)** M stage, **(J)** TNM stage, **(K)** surgery and radiotherapy, **(L)** chemotherapy.

**Figure 3:**
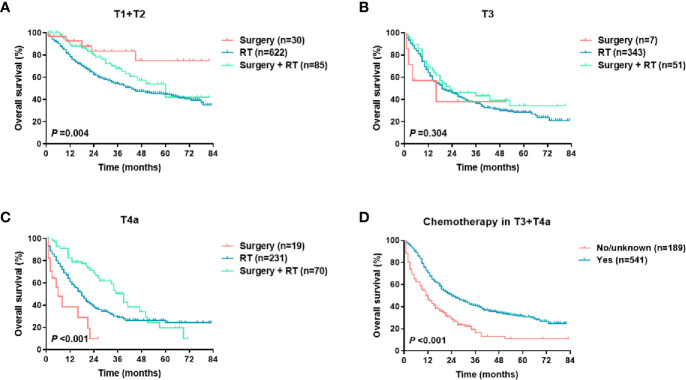
OS of metastasis-free HSCC patients in different treatment groups (per T stage): **(A)** local treatment for T1 and T2 disease; **(B)** local treatment for T3 disease; **(C)** local treatment for T4a disease; **(D)** chemotherapy in T3 and T4a patients.

For the training set, the Cox univariate regression analysis showed that the following parameters were significantly related to OS: age, marital status, race, insurance status, primary site, T stage, N stage, M stage, TNM stage, local treatment, and chemotherapy (**Table 2**). **Figure 2** shows the OS curves, which were based on the Kaplan-Meier method and log-rank test and accounted for the following parameters: age, marital status, race, insurance, primary site, pathological differentiation, T stage, N stage, M stage, TNM stage, local treatment, and chemotherapy. A competing risk analysis showed that age, marital status, race, T stage, N stage, M stage, local treatment, and chemotherapy were still correlated with HSCC-specific death (all P < 0.05, **Figure S1**). A subgroup analysis was also performed for T stage in patients with local resectable HSCC to analyze the relationship between local treatment and OS (**Figure 3**). In T3 patients, no significant difference was observed in OS among patients who received surgery alone, those who received radiotherapy alone, and those who received both surgery and radiotherapy (P = 0.304). In T4a patients, however, a significant between-group difference was observed in OS (P < 0.001), which was longest in patients who received both surgery and radiotherapy, followed by patients who received radiotherapy alone, and then patients who received surgery alone. Moreover, T3 and T4a patients who received systemic chemotherapy had a significantly longer OS than those who did not receive chemotherapy (P < 0.001). We further analyzed the overall survival of metastasis-free HSCC patients with different treatment modalities for each TNM stages, as shown in **Figure S2**. It was found that for locally advanced HSCC, the curative effect of single treatment modality was relatively poor, while that of combined therapy was relatively better (**Figures S2B–D**, P < 0.001).

**Table 2:** Univariate and multivariate analyses of overall survival in the training set.

Variable	Univariate analysis		Multivariate analysis	
	HR (95% CI)	*P* value^a^	HR (95% CI)	*P* value
Age				
≤50 years	Reference			
51-60 years	1.176 (0.921-1.503)	0.193	1.138 (0.887-1.459)	0.308
61-70 years	1.168 (0.915-1.49)	0.212	1.248 (0.972-1.603)	0.083
>70 years	1.671 (1.305-2.138)	**<0.001**	1.947 (1.503-2.523)	**<0.001**
Sex				
Male	Reference			
Female	0.879 (0.737-1.048)	0.152	NA	
Marital status				
Married	Reference			
Others	1.487 (1.304-1.696)	**<0.001**	1.344 (1.173-1.541)	**<0.001**
Race				
White	Reference			
Black	1.677 (1.433-1.962)	**<0.001**	1.333 (1.13-1.573)	**0.001**
Others	0.991 (0.768-1.277)	0.942	0.968 (0.748-1.252)	0.803
Insurance				
No/unknown	Reference			
Yes	0.676 (0.542-0.841)	**<0.001**	0.814 (0.648-1.021)	0.075
Primary site				
Pyriform sinus	Reference			
Postcricoid region	1.187 (0.795-1.774)	0.402	1.214 (0.809-1.82)	0.349
Posterior pharyngeal wall	1.161 (0.903-1.492)	0.245	1.243 (0.963-1.604)	0.095
NOS	1.225 (1.068-1.404)	**0.004**	1.155 (1.006-1.326)	**0.042**
Grade				
Well differentiated	Reference		NA	
Moderately differentiated	1.18 (0.824-1.691)	0.366		
Poorly differentiated	1.049 (0.729-1.509)	0.797		
Undifferentiated	0.259 (0.062-1.082)	0.064		
Unknown	1.022 (0.703-1.487)	0.908		
T stage				
T1	Reference			
T2	1.602 (1.206-2.129)	**0.001**	1.66 (1.244-2.215)	**0.001**
T3	2.407 (1.808-3.204)	**<0.001**	2.575 (1.919-3.454)	**<0.001**
T4a	2.678 (1.999-3.588)	**<0.001**	2.958 (2.194-3.989)	**<0.001**
T4b	3.725 (2.721-5.101)	**<0.001**	3.403 (2.461-4.706)	**<0.001**
N stage				
N0	Reference			
N1	1.232 (1.007-1.508)	**0.043**	1.389 (1.128-1.709)	**0.002**
N2	1.43 (1.213-1.685)	**<0.001**	1.679 (1.408-2.002)	**<0.001**
N3	2.282 (1.737-3)	**<0.001**	2.795 (2.084-3.749)	**<0.001**
M stage				
M0	Reference			
M1	3.455 (2.85-4.188)	**<0.001**	2.686 (2.191-3.293)	**<0.001**
TNM stage				
I	Reference		NA	
II	2.117 (1.166-3.843)	**0.014**		
III	2.469 (1.4-4.354)	**0.002**		
IVA	3.31 (1.907-5.744)	**<0.001**		
IVB	5.2 (2.946-9.178)	**<0.001**		
IVC	10.754 (6.06-19.085)	**<0.001**		
Surgery and radiotherapy				
Surgery	Reference			
Radiotherapy	1.125 (0.786-1.61)	0.520	1.242 (0.857-1.801)	0.252
Surgery+ radiotherapy	0.715 (0.479-1.068)	0.101	0.724 (0.48-1.092)	0.123
Both not given	3.056 (1.649-5.665)	**<0.001**	2.088 (1.117-3.901)	**0.021**
Chemotherapy				
No/unknown	Reference			
Yes	0.6 (0.524-0.686)	**<0.001**	0.484 (0.416-0.564)	**<0.001**

HSCC, hypopharyngeal squamous cell carcinoma; NOS, not otherwise specified; TNM, tumor-node-metastasis; HR, hazard ratio; CI, confidence interval.

^a^P values < 0.05 are indicated in bold.

**Figure 4:**
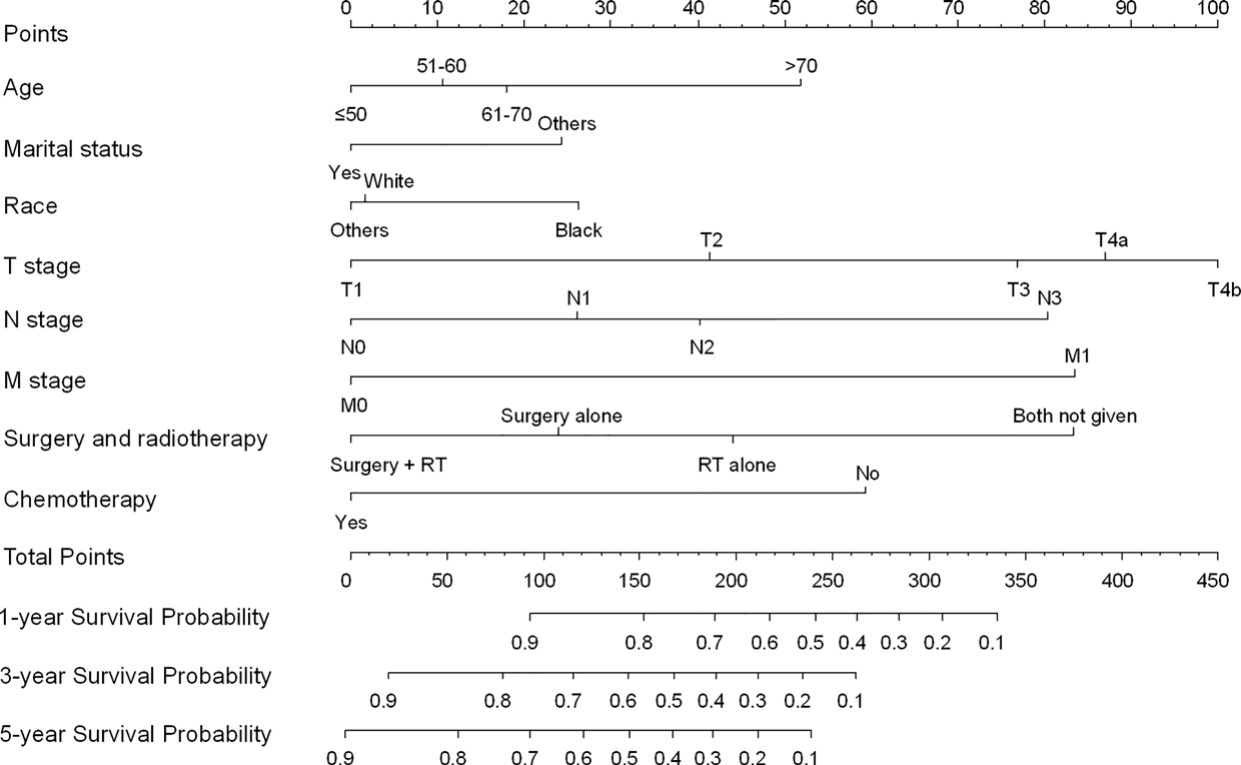
Nomogram for predicting the survival probability of HSCC patients.

A Cox multivariate regression analysis showed that age, marital status, race, T stage, N stage, M stage, local treatment, and chemotherapy were independent prognostic factors for OS (**Table 2**). TNM stage was excluded from the multivariate analysis because it was not an independent variable, but rather, it is a combination of T, N, and M stages. The eight significant independent prognostic factors (age, marital status, race, T stage, N stage, M stage, local treatment, chemotherapy; P < 0.05) identified by the Cox multivariate regression analysis were used to develop a prognostic nomogram (**Figure 4**). The score of each prognostic factor was as follows (in descending order): age > 70: 52; marital status - other: 24; race - black: 26; T4b: 100; N3: 80; M1: 83; surgery and radiotherapy (no): 83; and chemotherapy (no): 59. The total score was used to predict each patient’s 1-year, 3-year, and 5-year survival probabilities. For example, for a 65-year-old married Chinese patient diagnosed with HSCC T3N2bM0 who received radical chemoradiotherapy, the prognostic nomogram scored the age as 12, the marital status as 0, race as 0, T3 as 77, N2 as 40, M0 as 0, radiotherapy as 44, and chemotherapy as 0, which resulted in a total score of 173. Therefore, the model predicted that the 1-year, 3-year, and 5-year survival probabilities were 76%, 48%, and 37%, respectively.

### Validation of the Nomogram

**Figure 5:**
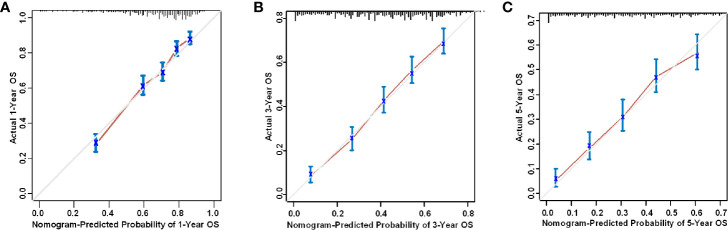
Internal calibration curve of the nomogram for the prediction of the **(A)** 1-year, **(B)** 3-year, and **(C)** 5-year survival probability of HSCC patients.

The nomogram was validated with both internal and external validation. For the internal validation, the calibration curve showed that the nomogram was accurate in its predictions (**Figure 5**). The X-axis represents the survival probability predicted by the nomogram, and the Y-axis represents the actual survival probability. The dotted line (45° diagonal line) indicates complete concordance between the actual probability and the predicted probability. The similarity between the solid line and the dotted line indicates a high level of accuracy in nomogram prediction. Next, the C-index and the Brier score were used to evaluate the performance of the prognostic nomogram, which was compared with that of the TNM staging system (**Table S2**). For the external validation, the patients in the validation set were rated with the nomogram, and then the total scores were incorporated into the Cox regression model to calculate the C-index. The C-index of the nomogram was greater than 0.7, which was higher than that of the TNM staging system (training set: 0.718 *vs* 0.627; validation set: 0.708 *vs* 0.598; external validation set: 0.709 *vs* 0.597). The nomogram also performed better than the TNM staging system as assessed by the Brier score (lower values indicate better model performance, **Table S2**). **Figure S3** shows that in the training set, the validation set, and the external validation set, the area under the curve (AUC) values for the 1-year, 3-year, and 5-year OS curves were higher for the nomogram than for the TNM staging system, which suggests that the nomogram is superior to the TNM staging system in predicting clinical outcomes.

**Figure 6:**
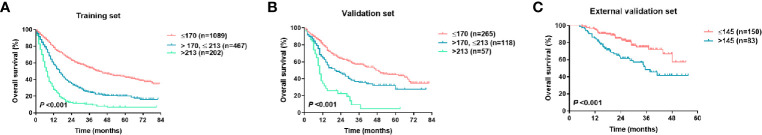
Kaplan-Meier survival curves of the training set **(A)**, the validation set **(B)**, and the external validation **(C)** per nomogram score.

Next, we divided the patients in the training and validation sets into the following three groups based on the 3-year survival probability predicted by the nomogram: the low-risk group (3-year survival probability ≥ 50%, score ≤ 170), the moderate-risk group (30% ≤ 3-year survival probability < 50%, 170 < score ≤ 213), and the high-risk group (3-year survival probability < 30%, score > 213). The Kaplan-Meier curve illustrates the good prognostic discrimination of the nomogram (P < 0.001, **Figures 6A, B**). The patients in the external validation group were divided into the following two groups based on the 3-year survival probability predicted by the nomogram: the low-risk group (3-year survival probability ≥ 50%) and the high-risk group (3-year survival probability < 50%). The survival curves confirmed a significant between-group difference (P < 0.001, **Figure 6C**).

## Discussion

Previous SEER-based studies have analyzed the tumor characteristics, treatment, and survival of patients with HSCC (6–8). However, other than the TNM staging system, a unified prediction model for HSCC is lacking due to the low prevalence of this disease. This is the first SEER-based study to develop a nomogram prediction model of HSCC survival. Heng Y et al. recently developed a prognostic nomogram for Chinese patients with HSCC after tumor resection, which served as a stratification indication for postoperative adjuvant treatment (13). However, the optimal initial treatment modality for locally advanced HSCC has not been fully defined and was identified as an important prognostic factor (6–8). Thus, in this SEER-based study, we analyzed the effect of local treatment (surgery and/or radiotherapy) and chemotherapy on OS. We also analyzed the prognostic factors of HSCC, developed an intuitive nomogram to effectively predict OS, and confirmed the validity of the prediction model using both internal and external validation. The nomogram may be used to evaluate the survival probability of each HSCC patient and provide a reference for the clinical assessment of patient outcomes and treatment strategies.

According to the revised TNM staging system presented in 2002 in the sixth edition of the AJCC Cancer Staging Manual, stage T4 can be further classified as T4a (moderately advanced local disease) or T4b (very advanced local disease). As a result, stage IV is further classified as stage IVA (moderately advanced local/regional disease), stage IVB (very advanced local/regional disease), and stage IVC (distant metastatic disease) (14). According to the 2010 seventh edition of the AJCC Cancer Staging Manual, HSCC-related esophageal involvement was revised from stage T4 (as described in the sixth edition) to stage T3 (15). In this study, we selected patients who were diagnosed in 2010 or later and who were staged according to the seventh edition of the AJCC Cancer Staging Manual. A survival analysis showed that the revised TNM stage was a good prognostic factor for HSCC (**Figures 2G–J**). However, we found limitations in the TNM stage. For example, the survival curve of stage T3 patients overlapped with that of stage T4a patients (**Figure 2G**), and no difference was observed in the OS prediction between stages II and III (**Figure 2J**), which suggests that the TNM staging prognostic system requires further improvement.

As in previous reports, this study showed that age was an important prognostic factor, and an age > 70 was associated with a more adverse prognosis (**Figure 2A**) (4, 5). In this SEER cohort, 23.8% of the patients were older than 70 at the time of HSCC diagnosis. The multivariate analysis showed that the hazard ratio was 1.947 (95% CI, 1.503-2.523, P < 0.001) in patients older than 70 relative to those aged 50 or below, in part because older patients tended to have more comorbidities and a shorter life expectancy and tended to receive more conservative treatment. The male: female ratio was approximately 5:1, which is similar to that reported in previous studies (4), but the univariate analysis revealed no difference in prognosis between the sexes. As shown in previous reports, this study reported that race, marital status, and insurance status were related to the OS of HSCC patients (**Figures 2B–D**), which to some extent reflected the effects of economic condition, social status, and emotional support on disease prognosis (16, 17). In this study, the univariate analysis showed that the primary site was a prognostic factor. “Primary site - not otherwise specified (NOS)” was associated with the worst prognosis, and no significant difference was observed in the prognosis of patients with tumors in other primary sites such as the piriform sinus, postcricoid region, and posterior pharyngeal wall (**Figure 2E**). In HSCC, it is often difficult to discern the primary site due to the large tumor size, which may explain the designation of “Primary site - NOS”. We also analyzed pathological differentiation and found that most cases were moderately differentiated and that pathological differentiation was unrelated to HSCC prognosis.

For HSCC, radiotherapy and surgery are important local treatments that are usually administered alone or in combination based on disease stage and pathological risk factors (such as positive margins and extracapsular involvement of lymph nodes) (2, 18–20). In the early stages of the disease, both treatments are viable options; in locally advanced-stage disease, surgery plus radiotherapy helps improve the local control rate and the prognosis. In this study, radiotherapy and surgery were analyzed as a composite variable. Our SEER data showed that in America, radiotherapy alone is the most common treatment modality for HSCC (80.8%), followed by surgery plus radiotherapy (14.1%). Consistent with previous studies (6–8), this study showed that local treatment patterns were independent prognostic factors for survival (**Table 2** and **Figure 2K**). A population‐based cohort study that involved 6647 HSCC patients showed that the best 5-year OS rate (48.5%) was achieved with a combination of surgery and radiation therapy. The 5-year OS rate of patients treated with surgery was significantly higher than that of those treated with radiotherapy alone in cases of local (63.3% *vs* 52.4%) or regionally advanced disease (41.3% *vs* 31.9%) (6). We then performed a subgroup analysis of T stage to determine the effect of local treatment on the survival of patients with HSCC without distant metastasis (**Figures 3A–C**). Among the HSCC patients with stages T1 and T2 disease, most received radiotherapy, although surgery alone was also effective (P = 0.004). Surgery plus radiotherapy was the best option for patients with stage T4a disease (P < 0.001), but this combination had no significant advantage in patients with stage T3 disease (P = 0.304). A population-based study in the Netherlands also indicated that overall survival of stage T3 patients was equal after total laryngectomy and (chemo)radiotherapy, but a survival benefit was achieved after primary surgery ± radiotherapy for T4 patients (18). In general, systemic chemotherapy improves HSCC prognosis (2, 7, 19). Our data demonstrated that chemotherapy significantly reduced mortality (HR 0.489, 95% CI 0.416-0.564, P < 0.001) (**Table 1** and **Figure 2L**), and this effect was even more pronounced in patients with locally advanced HSCC with stages T3 and T4 disease (**Figure 3D**).

In this study, eight prognostic factors were incorporated into our Cox multivariate analysis to develop a nomogram, including demographics (age, marital status, race, insurance status), clinicopathological parameters (primary site, T stage, N stage, M stage), and treatment (local treatment and chemotherapy). The selection of these parameters was reasonable, feasible, and practical. Further validation showed a high level of accuracy in the prediction ability of the nomogram, which was superior to that of the TNM staging system (**Figure S2**). Nevertheless, this study has some limitations. First, this is a SEER-based population cohort study. Patients with missing data were excluded from the study, which may have led to bias. Second, in the SEER database, chemotherapy was categorized as “No/Unknown” or “Yes”, with no details on modality, such as induction chemotherapy, concurrent chemotherapy, and adjuvant chemotherapy, and no details on the type or dose of chemotherapy drug, which may have led to information bias and may have affected the HR of the variables. Third, the SEER database does not include some of the known pathological prognostic factors for HSCC, such as positive margins or extracapsular involvement of lymph nodes. As a result, we were unable to incorporate these factors into the prediction model. Fourth, the SEER database provided OS and CSS data but not progression-free survival or local relapse-free survival data, which would have affected the survival prediction of the nomogram. Finally, the SEER database is based on the US population. Therefore, the nomogram may only serve as a reference for prognostic prediction in the Chinese HSCC population. In the future, large multicenter studies should be performed in Chinese patients to develop a prediction model for the Chinese population.

## Conclusion

This SEER-based study shows that some demographic characteristics, clinicopathological parameters, and treatment strategies are significantly correlated with the survival outcomes of HSCC patients. We developed and validated a nomogram for HSCC that had superior discrimination and accuracy. The variables are easy to collect, which demonstrates the ease of use of this nomogram in clinical practice to aid in the clinical evaluation of the risk level of HSCC patients and the development of individualized treatment strategies.

## Data Availability Statement

Publicly available datasets were analyzed in this study. This data can be found here: SEER database (https://seer.cancer.gov):Incidence -SEER 18 Regs Custom Data with additional treatment fields, Nov 2018 Sub, 1975-2016 varying.

## Ethics Statement

The studies involving human participants were reviewed and approved by The Institutional Review Board of the Eye Ear Nose and Throat Hospital, Fudan University. The patients/participants provided their written informed consent to participate in this study.

## Author Contributions

ST, XH, RL, and ZT conceived and designed the research. ST, QL, RL, and XC performed statistical analysis and analyzed the results. ST and HG followed the patients and collected clinical data. ST and XW wrote and revised the paper. All authors contributed to the article and approved the submitted version.

## Funding

This work was funded by National Science and Technology Major Project (2020ZX09201-013) and Science and Technology Commission of Shanghai Municipality (19411961300).

## Conflict of Interest

The authors declare that the research was conducted in the absence of any commercial or financial relationships that could be construed as a potential conflict of interest.
