# Supplementary material for: Development and Validation of a Prognostic Nomogram for Hypopharyngeal Carcinoma
Source: Front Oncol. 2021 Jun 21;11:696952. doi: 10.3389/fonc.2021.696952 (PMC8255987; doi:10.3389/fonc.2021.696952)
Supplement: Supplementary file 1 [file DataSheet_1.pdf]

Supplementary Material

1 Supplementary Figures and Tables

1.1 Supplementary Figures

Supplementary Figure 1. Competing risk analysis of HSCC patients in different subgroups: (A) age, (B) marital status, (C) race, (D) T stage, (E) N stage, (F) M stage, (G) surgery and radiotherapy, (H) chemotherapy. 1: cancer-specific death; 2: noncancer-specific death.

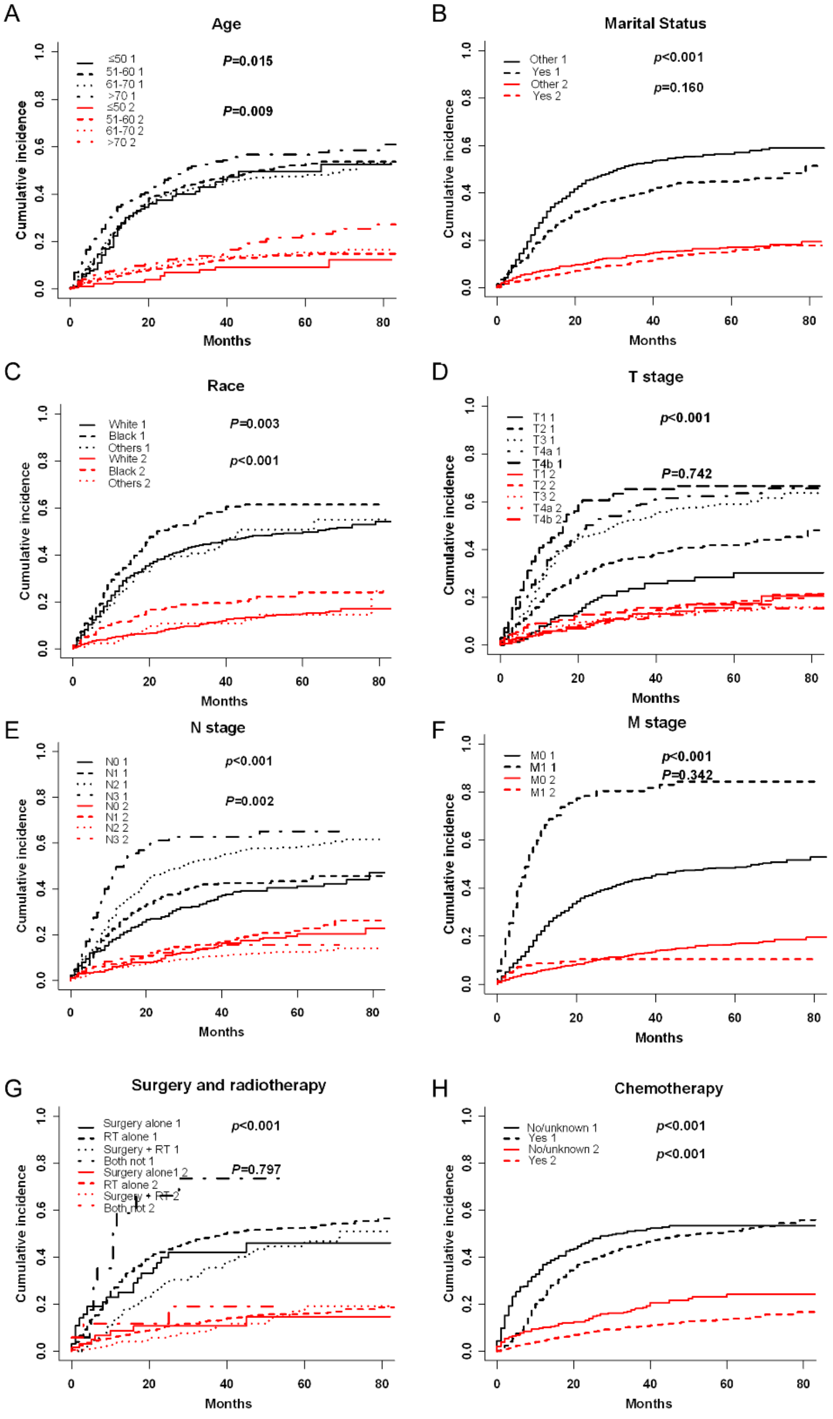

Supplementary Figure 2. OS of metastasis-free HSCC patients treated with different treatment modalities (per TNM stage): (A) Stage I and stage II; (B) Stage III; (C) Stage IVA; (D) Stage IVB. Abbreviations: RT, radiotherapy; CT chemotherapy.

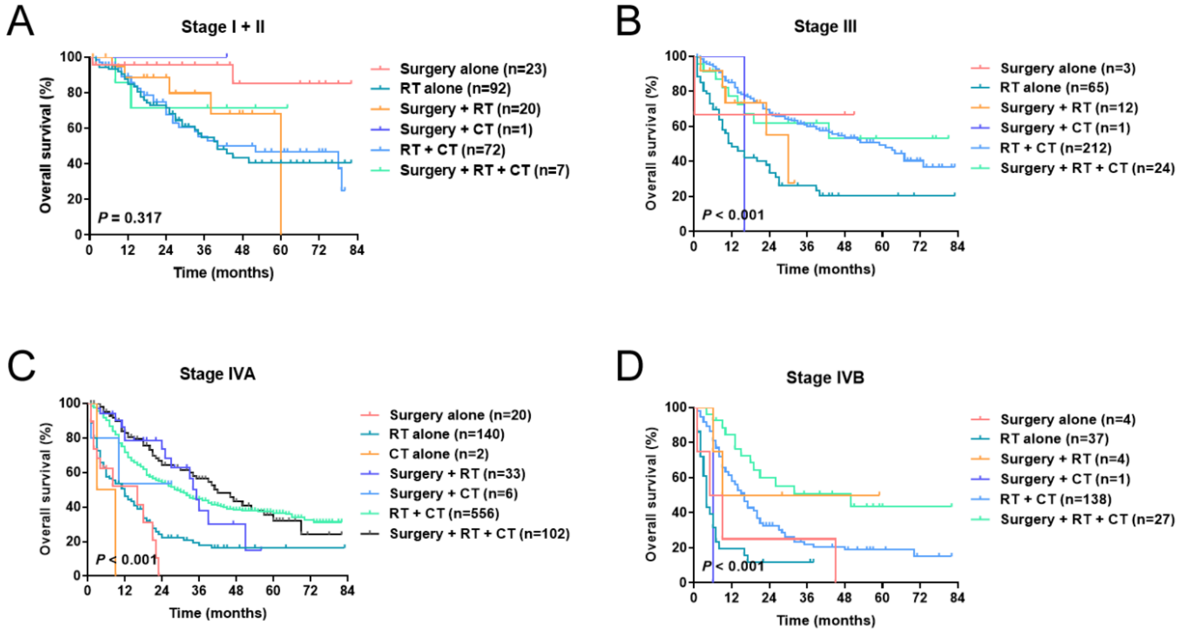

Supplementary Figure 3. AUCs of survival curves of patients in the training set (A-C), the validation set (D-F), and the external validation set (G-I) to compare the nomogram and the TNM staging system. (A), (D), (G): 1-year OS; (B), (E), (H): 3-year OS; (C), (F), (I): 5-year OS. The red line indicates the survival probability predicted by the nomogram; the blue line indicates the survival probability predicted by the TNM staging system.

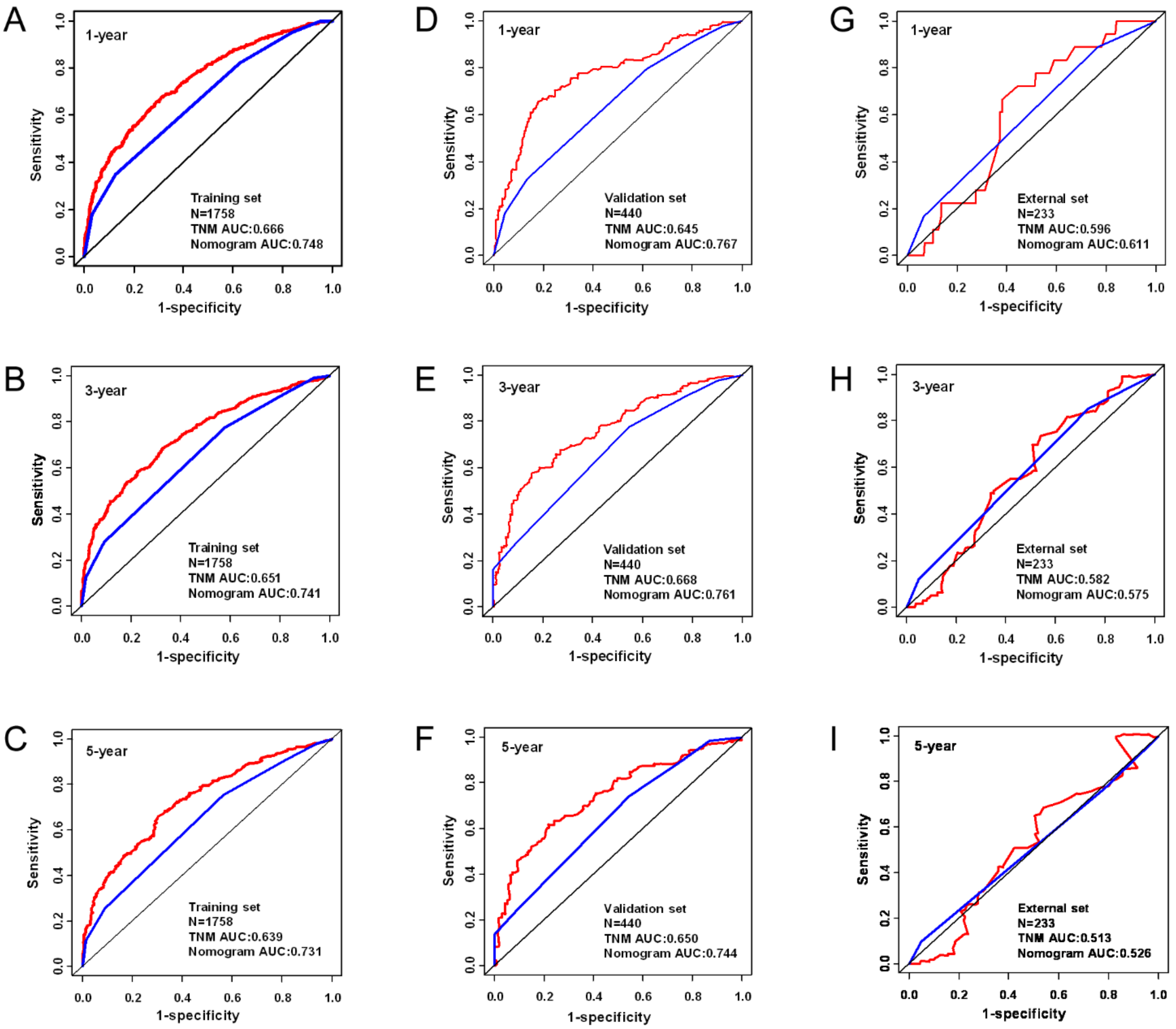

1.2 Supplementary Tables

Supplementary Table 1. Demographics and clinical characteristics of the 233 HSCC patients in the external validation set

Characteristic	Patients
Age	
≤50 years	29 (12.4)
51-60 years	86 (36.9)
61-70 years	100 (42.9)
>70 years	18 (7.7)
Sex	
Male	229 (98.3)
Female	4 (1.7)
Marital status	
Married	233 (100)
Others	0 (0)
Race	
White	0 (0)
Black	0 (0)
Others	233 (100)
Insurance	
No	0 (0)
Yes	233 (100)
Primary site	
Pyriform sinus	179 (76.8)
Postcricoid region	15 (6.4)
Posterior pharyngeal wall	39 (16.7)
NOS	0 (0)
Grade	
Well differentiated	12 (5.2)
Moderately differentiated	174 (74.7)
Poorly differentiated	28 (12)
Undifferentiated	0 (0)
Unknown	19 (8.2)
T stage	
T1	6 (2.6)
T2	65 (27.9)
T3	82 (35.2)
T4a	80 (34.3)
T4b	0 (0)
N stage	
N0	24 (10.3)
N1	69 (29.6)
N2	123 (52.8)
N3	17 (7.3)
M stage	
M0	233 (100)
M1	0 (0)
TNM stage	
I	0 (0)
II	0 (0)

III	52 (22.3)
IVA	164 (70.4)
IVB	17 (7.3)
IVC	0 (0)
Surgery and radiotherapy	
Surgery	0 (0)
Radiotherapy	51 (21.9)
Surgery + radiotherapy	182 (78.1)
Both not given	0 (0)
Chemotherapy	
No	40 (17.2)
Yes	193 (82.8)

Abbreviations: HSCC, hypopharyngeal squamous cell carcinoma; NOS, not otherwise specified; TNM, tumor-node-metastasis.

Supplementary Table 2. Comparison of the C-indexes and Brier scores between the nomogram and TNM staging in HSCC patients

	**C-index (95% CI)**	**Brier Score**
Training set		
Nomogram	0.718 (0.709-0.727)	0.179
TNM stage	0.627 (0.618-0.636)	0.198
Validation set		
Nomogram	0.708 (0.689-0.727)	0.180
TNM stage	0.598 (0.578-0.617)	0.201
External set		
Nomogram	0.709 (0.678-0.740)	0.196
TNM stage	0.597 (0.570-0.624)	0.225
